# Role of Type and Volume of Recreational Physical Activity on Heart Rate Variability in Men

**DOI:** 10.3390/ijerph17082719

**Published:** 2020-04-15

**Authors:** Shaea Alkahtani, Andrew A. Flatt, Jawad Kanas, Abdulaziz Aldyel, Syed Shahid Habib

**Affiliations:** 1 Department of Exercise Physiology, College of Sport Sciences and Physical Activity, King Saud University, Riyadh 11451, Saudi Arabia; Jawad-Kanas@outlook.sa (J.K.); aldayel@ksu.edu.sa (A.A.); 2 Department of Health Sciences and Kinesiology, Biodynamics and Human Performance Center, Georgia Southern University – Armstrong, Savannah, GA 31419, USA; aflatt@georgiasouthern.edu; 3 Department of Physiology, College of Medicine & King Khalid University Hospital, King Saud University, Riyadh 11461, Saudi Arabia; sshahid@ksu.edu.sa

**Keywords:** sedentary, walking, cycling, time and frequency domains, blood pressure

## Abstract

The aim of this study was to investigate the effect of recreational aerobic physical activity (PA) type and volume on heart rate variability (HRV) in Arab men. This was a retrospective, cross-sectional study, and included men (*n* = 75, age = 37.6 ± 7.1 years, body mass index (BMI) = 26.7 ± 3.1 kg/m^2^) who were members of a walking group, cycling group, or were inactive controls. Monthly distances from the past three months were obtained from walking and cycling groups, and the volume of PA was classified into three subgroups (high, moderate, low). HRV was measured using a computerized electrocardiographic data acquisition device. R–R interval recordings were performed while participants rested in a motionless supine position. RR intervals were recorded for 15 minutes, and a five-minute segment with minimal ectopic beats and artifacts was selected for HRV analysis. Time-domain parameters included the mean R–R interval, standard deviation of the mean R–R interval (SDNN), and root-mean-squared difference of successive RR intervals (RMSSD). The frequency-domain parameters included high-frequency power (HF), low-frequency power (LF), and LF to HF ratio (LF/HF). Results showed that there were no significant differences between walking, cycling, and control groups for all HRV parameters. Time-domain analyses based on PA volume showed that age-adjusted SDNN for the high-active group was greater than the low-active group (*P* = 0.03), and RMSSD for the moderate-active group was greater than the control group (*P* = 0.009). For the frequency domain, LF for the high-active group was greater than the low-active and control groups (*P* = 0.006), and HF for the moderate-active group was greater than the low-active group (*P* = 0.04). These data indicate that walking >150 km per month, or cycling >100 km per month at a speed >20 km/h may be necessary to derive cardiac autonomic benefits from PA among Arab men.

## 1. Introduction

Heart rate variability (HRV) is a valid marker that reflects cardiac modulation by sympathetic and parasympathetic components of the autonomic nervous system (ANS) [1]. The clinical applications of HRV are mainly associated with the prediction of sudden cardiac death and assessing the progression of cardiovascular and metabolic conditions [2]. Indeed, lower vagal-related HRV indices are associated with elevated markers of inflammation, blood lipids and triglycerides, blood pressure, and blood glucose [3]. Computerized electrocardiographic (ECG) recordings are typically used to monitor HRV. Cardiac cycle length (R–R interval) in relation to the immediately preceding R–R interval can be converted to a scatter plot. Using specialized software, artifacts and ectopic beats are filtered prior to computation of time- and frequency-domain parameters of HRV. These methods of evaluating HRV are generally accepted for reflecting cardiac parasympathetic nervous system activity (PNSA) [4]. Interactions between sympathetic and parasympathetic activity can be assessed by low-frequency power/high-frequency power ratio (LF/HF) [2]. Low-frequency power (LF) from 0.04 to 0.15 Hz is related to baroreceptor sensitivity mediated by vagal and sympathetic systems, whereas high-frequency power is influenced by respiratory sinus arrhythmia [5].

Physical activity (PA) guidelines for adults consider the volume of PA performed at low–moderate intensities, which improves many health parameters [6]. Whether such guidelines are applicable for improving HRV has been the topic of recent investigation. While high-intensity training has been shown to improve HRV, findings pertaining to the effects of low- and moderate-intensity PA are inconsistent. For example, the use of high-intensity interval exercise performed at maximal, near-maximal, or supramaximal aerobic power may have a greater effect on HRV [7]. Another study found that high-intensity interval training at 130% of maximal oxygen uptake (VO_2max_) for three weeks increased HRV threshold [8]. On the other hand, training at moderate intensity levels for 24 sessions increased (VO_2max_) by 11% among sedentary middle-age men, but did not increase vagal modulation as measured using HRV [9]. Similar outcomes were observed after an eight-week exercise intervention among old adults [10]. Factors such as age and duration of the intervention were hypothesized to explain these findings [1]. Thus, while athletes can safely train at ≥ maximal intensities, which improves HRV [11], there are many precautions for performing high-intensity exercise for the general public [6]. This highlights a need for examining the role of low–moderate-intensity PA on HRV, which remains inconclusive. 

Aerobic training such as walking and cycling are the predominant modes of PA among active groups with voluntary membership in Saudi Arabia. However, key variables for improving HRV such as intensity and duration of these free-living activities are rarely well-supervised [12]. A six-month randomized active-controlled trial showed that structured PA can improve HRV to a greater extent than unstructured PA in adolescents [13]. Therefore, recreationally active groups may be less consistent than athletes in performing PA, which may limit the positive effects of training on HRV. Ethnicity is an additional factor that may influence HRV, with possible implications for metabolic and psychological outcomes [14,15]. With a lack of studies investigating HRV and PA among Saudis and Arabs [16], it is important to examine how HRV is affected by regular recreational PA among this underrepresented population.

Given the association between HRV and various health markers, further investigation into modifiable factors that can potentially improve HRV is warranted. Performing more PA is a potential lifestyle intervention that individuals can adopt to improve HRV, although how factors such as type and volume of PA effect cardiac autonomic function, particularly among individuals of Saudi-Arabian descent, requires further investigation. Therefore, the current study aimed to examine the effect of recreational aerobic PA type and volume on HRV in Arab men living in Saudi Arabia. We hypothesized that men who performed regular walking and cycling would exhibit higher levels of HRV compared to age- and body composition-matched inactive men. A secondary hypothesis was that higher volumes of PA would be associated with greater HRV, independent of PA type. 

## 2. Materials and Methods 

### 2.1. Study Design 

This was a retrospective cross-sectional study. HRV parameters were dependent variables and the volume and type of PA were independent variables. 

### 2.2. Participants

**Figure 1:**
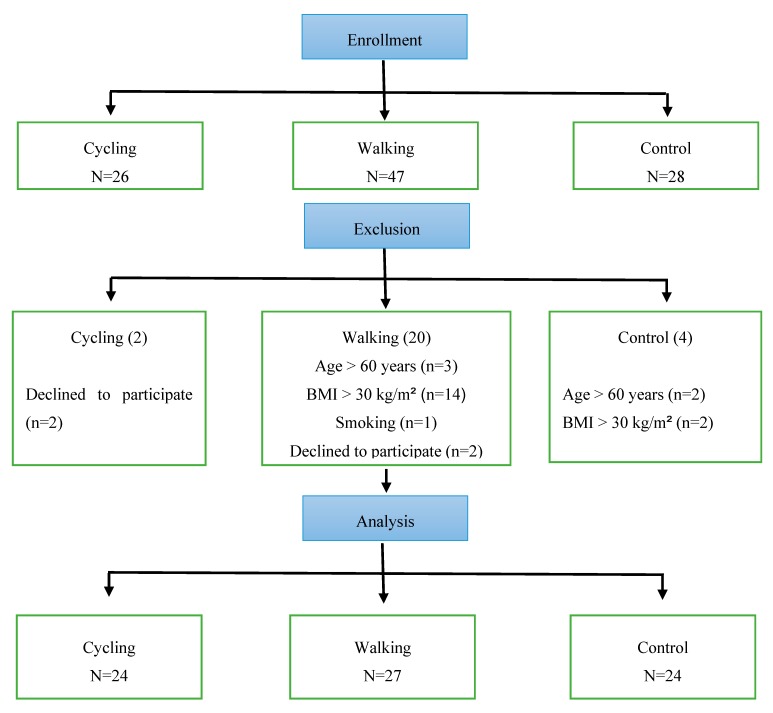
Flowchart of participants’ recruitment and exclusion process.

Study participants included men (*n* = 75, age = 37.6 ± 7.1 years, body mass index (BMI) = 26.7 ± 3.1 kg/m^2^, fat percent = 24.1% ± 5.4%) without any chronic medications and with normal resting heart rate (HRrest), and systolic (SBP) and diastolic (DBP) blood pressure (HRrest = 59.7 ± 6.0 b·min^−1^, SBP = 115.3 ± 14.2 mmHg, DBP = 82.6 ± 13.2 mmHg). Participants were members of the general public and were categorized according to PA type which included a walking group (Riyadh Walkers), a cycling group (Saudi Cyclists), and an inactive control group. To meet inclusion criteria, it was required that volunteers must: have engaged in regular PA for the past 3 months (for walking and cycling groups); be non-smokers; be <60 years old; have a BMI <30 kg/m^2^; and be free from injury or a newly diagnosed chronic illness within the past year. Figure 1 shows the flowchart of participants’ inclusion/exclusion in the study. Participants voluntarily expressed their interest by contacting the principal researcher or research assistant. Study aims and instructions for participation were provided by the researchers to prospective individuals. All participants signed a consent form of voluntary participation. This study was approved by the institutional review board (IRB) of King Saud University (IRB No. E-18-3381) and conformed with the guidelines provided by the Declaration of Helsinki.

### 2.3. Pre-Screening Procedures

Study measures were obtained in the Exercise Physiology Laboratories within the Department of Exercise Physiology at the College of Sport Sciences and Physical Activity at King Saud University. All participants were pre-screened for any cardiac abnormalities related to rhythm, ischemia, or heart size with a standard 12 lead ECG prior to HRV acquisition. All subjects that were included in the study had normal resting ECG. SBP and DBP were measured using an automatic brachial sphygmomanometer (Omron HEM-7121, Omron Healthcare manufacturing, Japan).

Participants were instructed to maintain their habitual lifestyle and declare if they experienced greater than usual stress during the testing period. They were also asked to limit excess caffeine consumption and refrain from exercising 24 h prior to testing. Data collection took place early in the morning, at least 4 h post-prandial, and before interacting in their daily life commitments. Study measures included body composition and HRV. 

### 2.4. Study Variable Measures

Study variables included laboratory measures and field measures. Laboratory measures included body composition and HRV, whereas field measures included self-monitored distances of walking and cycling. 

#### 2.4.1. Body Composition

Height was measured to the nearest 0.1 cm using a stadiometer (Seca 213, Seca GmbH & Co., Hamburg, Germany), and body weight was measured to the nearest 0.1 kg using a digital scale (PD100 ProDoc, Detecto Scale, Cardinal, Webb City, MO, USA). Body mass index (BMI) was calculated by dividing body weight in kg by height in square meters. Body composition was measured using multi-frequency bioelectrical impedance analysis (BIA) (MC-980MA, Tanita Corporation, Tokyo, Japan) where participants stood barefoot on the scale while holding the handles for approximately 60 sec. Upon completion of the test, body fat percentage (BF%) was recorded for analysis.

#### 2.4.2. Heart Rate Variability 

HRV parameters were measured using a computerized ECG data acquisition device with 16 analog input channels (PL3516 PowerLab 16/35, ADInstruments Pty Ltd. New South Wales, Australia). R–R interval filtering of artifacts and ectopic beats and computation of time- and frequency-domain parameters was performed using customized software (LabChart v. 8.1.13 Windows, ADInstruments Pty Ltd. New South Wales, Australia). Time-domain parameters included the mean R–R interval, standard deviation of the mean R–R interval (SDNN), and root-mean-squared difference of successive RR intervals (RMSSD). The frequency-domain parameters included high-frequency power (HF) from 0.15 to 0.40 Hz, low-frequency power (LF) from 0.04 to 0.15 Hz, and LF to HF ratio (LF/HF).

R–R interval recordings were performed in a quiet room with dim lighting while participants rested in a motionless supine position. Use of electrical devices (e.g., smartphones) was prohibited during ECG recordings. Three electrodes were applied on the chest following the Einthoven triangle. Leads were connected from the device to the electrodes to record HRV. A trained instructor supervised the procedure and did not talk with the participants during the test. Participants were asked to breathe naturally and avoid swallowing during the recording. Following a 5-minute stabilization period, RR intervals were recorded for 15 minutes while the researcher monitored signal quality. From the 15-minute recording, a 5-minute segment with minimal ectopic beats and artifacts was selected for HRV analysis. 

#### 2.4.3. Volume of Physical Activity for Walking and Cycling Participants

PA volume was measured with smartphone tracking applications used by the walking (i.e., Nike Inc.) and cycling (i.e., Strava Inc.) groups. Walkers and cyclists were members of private communication forums within the applications where they announced their monthly distances to encourage less-active members to maintain and improve their PA levels. This strategy had been implemented among the groups for a year prior to the current study. The average distance from the past 3 months of 51 participants was automatically obtained from the application by walking and cycling group supervisors. Classification of PA volume was standardized independently for walking and cycling groups to account for the different modalities. The volume of PA for walking was divided into 3 levels as follows: low (50–<150 km/month), moderate (150–<300 km/month), and high (≥300 km/month). Whereas the volume of PA for cycling participants was divided into 3 levels as follows: low (50–<100 km/month, at average speed 17 ± 2 km/h), moderate (100–<300 km/month, at average speed 23 ± 2 km/h), and high (>400 km/month, at average speed 30 ± 4 km/h).

### 2.5. Statistical Analysis

Data were analyzed using SPSS (version 21, IBM). Continuous data were presented as mean ± standard deviation (SD) for normally distributed variables, and as median and (25th and 75th) percentiles for non-normal variables. All continuous variables were checked for normality using the Kolmogorov–Smirnov test. Logarithmic transformations were applied to non-normal variables. Univariate analysis of variance (ANOVA) was used to determine the effect of PA type (walking vs. cycling vs. control) and volume (high vs. moderate vs. low vs. control) on HRV parameters. If known confounders, such as age or BF%, differed between groups, analysis of covariance (ANCOVA) was performed using the significant confounder (e.g., age of BF%) as a covariate. The estimate of effect size was assessed for all parameters. *P*-values < 0.05 were considered statistically significant.

## 3. Results

Results based on activity type are presented in Table 1. There were no significant differences between groups for age or BF%. Although mean values for all time- and frequency-domain HRV parameters were higher for walking and cycling groups relative to the control group (or lower for LF/HF), there were no statistically significant differences between groups.

Results based on PA volume are presented in Table 2. There was no significant effect for BF%. However, age significantly differed between groups. Therefore, age-adjusted values were determined for HRV parameters. The time-domain analysis showed that after controlling for age, SDNN for the high-active group was significantly greater than the low-active group (*P* < 0.05). In addition, RMSSD for the moderate-active group was significantly greater than the control group (*P* < 0.01). For the frequency domain, LF for the high-active group was significantly greater than the low-active and control groups (*P* < 0.01). Additionally, HF for the moderate-active group was significantly greater than the low-active group (*P* < 0.05). There were no significant differences between the moderate- and high-active groups or between the low- and inactive control groups for all variables. No between-group differences were observed for the R–R interval or LF/HF ratio (*P* > 0.05).

## 4. Discussion

This study evaluated differences in HRV based on the type and volume of PA among Arab men. In disagreement with our first hypothesis, there were no significant differences between walking, cycling, and inactive control groups for all indices of HRV. In agreement with our second hypothesis, significant differences were observed between groups based on PA volume, independent of known confounders such as age and body fat. These findings suggest that the volume of PA could be an important determinant of HRV in recreationally active Saudi-Arabian men, regardless of PA type.

Unexpectedly, HRV parameters did not differ between active and inactive groups (Table 1). Conflicting findings have been reported within the available literature. For example, some studies have shown no differences in HRV between active and in active groups [17,18], while others found differences favoring active groups [19,20]. Some investigations have reported that even low PA can improve HRV to a greater extent than being sedentary. For example, a cross-sectional study of 84 adults suggested that aerobic exercise increased vagal HRV for all levels of PA [21]. Another investigation compared HRV between a sedentary group and two middle-aged groups with equivalent weekly PA energy expenditure, but different intensities. They found that HRV indices were greater in the two active groups compared to the sedentary group [20]. A recent study showed that even slight increments in PA are beneficial for cardiac autonomic regulation among young men, as RMSSD significantly increased according to PA categories from low, moderate, high, to highest. The multivariable linear regression analysis showed a significant positive relationship between self-reported PA and Ln-RMSSD, independent of BMI, waist circumference and BF% [22]. In short, we found no differences between active and inactive groups, which may be explained by the considerable heterogeneity in PA volume among active groups, which was subsequently found to account for differences in HRV (Table 2). In addition, despite being described as inactive controls, this group exhibited similar BF% values relative to both walking and cycling groups (Table 1). This may indicate comparable metabolic health profiles among groups, which may also help explain a lack of between-group differences in HRV [23]. 

As hypothesized, we observed significant between-group differences in HRV based on PA volume classifications (Table 2). This finding is in agreement with several previous studies. For example, a systematic review reported positive associations between moderate-to-vigorous PA and RMSSD [24]. In a study that measured HRV over five consecutive days in 37 men with a mean age of 33 years, participants were categorized as having low, medium, or high self-reported PA. Significantly higher HRV (Ln-RMSSD, R–R interval) was observed in the moderate- and high-PA groups compared with the low PA group [19]. Similarly, we found that recreationally moderate- and high-PA groups showed comparable levels of time- and frequency-domain HRV. Likewise, inactive and low-active groups showed comparable levels of time- and frequency-domain HRV, which were significantly lower than either moderate- or high- active groups. 

Our findings indicate that walking up to 150 km per month, or cycling up to 100 km per month at a speed slower than 20 km/h, were insufficient for improving HRV in the current population. This finding suggests that a PA volume- or intensity-related threshold likely exists that must be met to improve HRV. In support of this postulation regarding intensity, a recent investigation demonstrated a dose–response relationship for the effects of very vigorous PA (>8 metabolic equivalents) on HRV in 1040 adult men and women [25]. Participants were stratified into quintiles according to min per week of very vigorous PA. Group 5 was greater than Groups 1–3, but did not differ from Group 4 in any HRV indices, suggesting similar effects at the top quantiles of very vigorous PA [25]. The volume of PA that improves physical fitness and health markers such as body composition and blood pressure has been described based on pedometer-derived daily step counts [26], or intensity and duration of PA per week (e.g., 75 to 150 minutes of high-intensity PA and 150 to 300 minutes of moderate-intensity PA) [27]. However, the volume of PA that improves HRV is less clearly defined. Thus, further research is needed to determine volume-based thresholds for PA that increases HRV, particularly for members of the population where higher intensity exercise may be contraindicated.

The main limitation of the current study was that we did not measure the intensity of walking. This factor has been addressed in many previous studies. For example, a five-year follow up from a large prospective study showed that walking distance and pace were positively associated with higher SDNN and ultra LF power [28]. It was also found that walking pace may contribute to less erratic sinus patterns and lower cardiovascular mortality, whereas general total leisure activity time did not show a similar association [28]. In another investigation, leisure-time PA, quantified as the hours of metabolic equivalent per week (MET.hr/week), and five-minute HRV were recorded for adults. There were significant linear trends of higher LF power with the highest quartile of vigorous activity, and lower HR with increasing quartile of moderate activity, suggesting that vigorous activity showed a possible mechanism by which PA reduces coronary heart disease risk [29]. 

In conclusion, the current study found that HRV among regular walking and cycling groups did not significantly differ from age- and body fat-matched inactive controls. However, when stratified by PA volume, greater HRV parameters were observed among moderate- and high-PA groups. From a practical perspective, the current findings indicate that walking >150 km per month, or cycling >100 km per month at a speed >20 km/h, may be necessary to derive cardiac autonomic benefits from PA. While further investigation is needed to identify more specific volume-related thresholds for improving HRV, these PA benchmarks may provide practical aiming points for healthy adult men seeking to improve cardiovascular health.

## Figures and Tables

**Table 1:** Comparison between physical activity types (walking, cycling, non-active control) for time- and frequency-domain parameters.

Parameters	Walking	Cycling	Non-Active	Effect Size	*P*-Value
N	27	24	24		
Age	38.0 ± 8.4	39.3 ± 7.5	35.5 ± 4.4	0.047	0.180
Fat (%)	24.4 ± 6.1	22.4 ± 4.9	25.7 ± 4.6	0.061	0.105
Ln-RR interval (sec)	1.05(0.91–1.12)	1.06 (1.0–1.1)	0.99 (0.92–1.1)	0.035	0.292
Ln-SDNN (ms)	53.3(31.1–69.9)	52.6(35.7–73.8)	45.8(37.9–54.9)	0.017	0.563
Ln-RMSSD (ms)	47.5(26.9–71.2)	39.4 (31.9–60.4)	35.2 (22.1–46.5)	0.039	0.250
Ln-LF (ms^2^)	918(246–2110)	764(230–1838)	594(414–1069)	0.009	0.765
Ln-HF (ms^2^)	786(310–2444)	609(345–1352)	408(223–1937)	0.038	0.317
Ln-LF/HF ratio	0.88(0.45–1.2)	0.76 (0.53–1.3)	1.07 (0.70–2.01)	0.025	0.464

**Note**: Data presented as mean ± SD for normal variables or median (25th–75th) percentile for non-normal variables; *P*-values < 0.05 considered significant.

**Table 2:** Comparison of physical activity (PA) volume between groups (low-, moderate-, high-, and non-active control) for time- and frequency-domain parameters.

Parameters	Volume of Physical Activity				Effect Size	*P*-Value	Adjusted for Age
	Low	Moderate	High	Control			
N	13	24	14	24			
Age	33.3 ± 9.3	40.0 ± 7.4^A^	41.1 ± 5.0^A^	35.5 ± 4.4	0.174	0.003	
Fat (%)	23.4 ± 7.0	23.9 ± 4.9	22.6 ± 5.6	25.7 ± 4.6	0.054	0.349	0.279
Ln-RR interval (sec)	1.0 (0.9–1.1)	1.08 (0.9–1.1)	1.06 (1.01–1.12)	0.99 (0.92–1.08)	0.096	0.074	0.201
Ln-SDNN (ms)	41.9 (30.9–63.3)	52.9 (32.2–69.9)	67.3 (44.1–77.9)^A^	45.8 (37.9–54.9)	0.079	0.135	0.025
Ln-RMSSD (ms)	32.6(18.6–52.9)	47.7 (35.3–69.6)	44.0 (32.5–73.1)	35.2 (22.1–46.5)^B^	0.066	0.198	0.009
Ln-LF (ms^2^)	338.7(196.6–764)	790 (203–2110)	1759(922–4942)^A^	594 (414–1069)^C^	0.183	0.006	< 0.001
Ln-HF (ms^2^)	481 (151.7–832)	676 (365–4347)^A^	838 (391–3109)	406 (223–1189)	0.163	0.014	0.004
Ln-LF/HF ratio	0.73 (0.46–1.18)	0.75 (0.40–0.95)	1.19 (0.59–2.1)	1.07 (0.70–2.01)	0.103	0.092	0.096

**Note**: Data presented as mean ± SD for normal variables or median (25th–75th) percentile for non-normal variables; *P* < 0.05 was considered statistically significant. ^A^ represents a significant difference relative to the low-active group; ^B^ represents a significant difference relative to the moderately-active group; and ^C^ represents a significant difference relative to the high-active group.
